# Hospital readmission with *Clostridium difficile* infection as a secondary diagnosis is associated with worsened outcomes and greater revenue loss relative to principal diagnosis: A retrospective cohort study

**DOI:** 10.1097/MD.0000000000012212

**Published:** 2018-09-07

**Authors:** Marya D. Zilberberg, Brian H. Nathanson, Stephen Marcella, John Jay Hawkshead, Andrew F. Shorr

**Affiliations:** a Evi*Med* Research Group, LLC, Goshen; b University of Massachusetts, Amherst; c OptiStatim, LLC, Longmeadow, Massachusetts; d Merck & Co, Inc, Rahway, New Jersey; e Washington Hospital Center, Washington, DC.

**Keywords:** *C difficile*, hospital costs, mortality, outcomes, rehospitalization

## Abstract

Recurrent *Clostridium difficile* infection (rCDI) requiring rehospitalization contributes to poor outcomes, which may differ between patients hospitalized with versus for it.

We performed a multicenter retrospective cohort study of rehospitalized adults surviving initial CDI hospitalization. Hospital mortality, length of stay (LOS), 30-day readmission, and mean gap between hospital costs and Diagnosis Related Group (DRG) reimbursement served as outcomes.

Among the 25.7% (n = 99,175) survivors requiring rehospitalization, 36,504 (36.8%) had rCDI (14,005 [38.4%] principal diagnosis rCDI [PrCDI]). Compared with non-CDI, PrCDI, and secondary diagnosis rCDI [SrCDI] carried lower risk of death (PrCDI odds ratio [OR] 0.52; 95% confidence interval [CI] 0.46, 0.58; SrCDI OR 0.80; 95% CI 0.75, 0.85) and 30-day readmission (PrCDI OR 0.84; 95% CI 0.80, 0.88; SrCDI OR 0.97; 95% CI 0.94, 1.01), and excess LOS (PrCDI 1.8 days; 95% CI 1.7, 2.0; SrCDI 1.4 days; 95% CI 1.3, 1.5), and costs (PrCDI $1399; 95% CI $858, $1939; SrCDI $2809; 95% CI $2307, $3311). Mean gap between hospital costs and DRG reimbursements was highest in SrCDI ($13,803).

A rehospitalization within 60-days of an initial CDI hospitalization occurs in approximately 25% of all survivors, 1/3 with rCDI. SrCDI carries worse outcomes than PrCDI. The potential loss of revenue incurred by the hospital is nearly 3-fold higher for SrCDI than PrCDI.

## Abstract 2

Supplemental Digital Content is available in the text

## 1 Introduction

Although clinical trials suggest that certain therapies can reduce recurrent *Clostridium difficile* infection (rCDI) rates by ≥50%, the condition remains both prevalent and burdensome.^[1–4]^ Reasons include the increasing complexity of patients potentially at risk for rCDI, the suboptimal use of the newer therapies, and the general differences between the quasi-artificial construct of clinical trials and real-world clinical practice.^[5–7]^ Regardless of the underlying reasons, rCDI continues to vex clinicians, torment patients, and affect hospitals.

Patients who have survived an initial hospitalization with CDI and then require a rehospitalization with a recurrence consume a disproportionate share of healthcare resources. In a cohort of such Medicare patients, we noted a recurrence rate of 33%, of whom 2/3 required a readmission to the hospital within a short interval.^[4]^ Although rCDI did not raise the risk of death in these subjects, the associated excess hospital days and costs were considerable.

While studies conflict as to whether rCDI increases mortality, its economic burden is not in question. What is less certain is what drives this burden.^[4,8–11]^ For example, there may be differences between patients who are admitted explicitly for the treatment of their rCDI and those who are admitted with another condition, where rCDI is a concurrent but not primary affliction. It is likely that those hospitalized *for* rCDI differ in important and potentially actionable ways from those hospitalized *with* rCDI. It is also unclear how much reimbursements for rCDI hospitalizations compare with the actual costs hospitals incur caring for these patients, as it is possible that the lengths of stay for patients with rCDI readmission exceed standard payments for these admissions. Finally, as the US healthcare system moves to a “value based” purchasing approach with a diminished willingness of payors to cover costs associated with readmissions, it is important to understand the current rates of 30-day readmissions among patients with a rCDI.^[12]^

## 2 Methods

### 2.1 Study design

We performed a retrospective cohort study to explore the epidemiology and outcomes of adult (age ≥18 years) rehospitalizations with rCDI in the United States. Outcomes of interest were hospital mortality, length of stay (LOS), hospital costs, 30-day readmission rates, and the reimbursements gap (defined below).

### 2.2 Data sources

We analyzed the State Inpatient Databases (SID), a part of the Health Care Utilization Project (HCUP) administered by the Agency for Healthcare Research and Quality (AHRQ), from 4 geographically diverse US states (California [only available for years 2009–2011], Florida, Iowa, and New York) for years 2009 to 2013.^[13]^ For further details on SID, please, view Supplemental material.

The 4 states representing each of the 4 Census Bureau regions of the United States were chosen because their geographic diversity increases the generalizability of our findings.^[14]^

### 2.3 Case identification

An initial CDI hospitalization episode was defined as a discharge with a CDI International Classification of Diseases, Ninth Revision, Clinical Modification (ICD-9-CM) code (008.45) between March 1 and October 31 of any year and no CDI codes during any hospitalization within prior 60 days. All rehospitalizations within 60 days following initial CDI discharge were included in the cohort. Those whose rehospitalizations included a CDI code were assigned to the rCDI group.^[4]^ Admissions with rCDI were subdivided into those whose rCDI was listed as the principal diagnosis (PrCDI), connoting the primary reason for admission, and those with rCDI as a secondary diagnosis (SrCDI), where rCDI was incidental to the hospitalization. For further definitions of rCDI categories (Community-onset healthcare facility associated [CO-HCFA], hospital-onset healthcare facility associated [HO-HCFA], and community-acquired [CA]), please, see Supplemental material.^[15]^

### 2.4 Follow-up

The groups were followed until in-hospital death or discharge from the hospital. For the 30-day readmission outcome, the survivors of the index rCDI hospitalization were followed for an additional 30 days with the sole purpose of quantifying this outcome.

### 2.5 Outcome variables

We examined mortality, hospital LOS in days, hospital costs in $US, as well as 30-day readmission rates among survivors of the readmission. HCUP collects hospital charges and provides Center for Medicare and Medicaid Services-defined individual hospital cost-to-charge conversion ratios as a part of the National Inpatient Database. Comparator groups were those readmitted with no evidence of rCDI (non-rCDI), those with PrCDI, and SrCDI. In addition, we explored the mean reimbursement gap between the top 5 Medicare Severity Diagnosis Related Groups (MS-DRG) within each comparator group (see below).^[16]^ While hospital costs represent a fixed fraction of that which is billed by the hospital for a service (charge), a DRG reflects the actual amount reimbursed to the hospital by the payor. The gap between costs and reimbursements may also be related to further negotiated discounting or penalties and rewards based on value delivered.

### 2.6 Statistical analyses

We compared demographic, clinical, hospital, and discharge characteristics among the 3 groups, as well as their outcomes—mortality, LOS, costs, and 30-day readmission rates. Differences between mean MS-DRG reimbursements and mean costs for the top 5 MS-DRGs in each of the comparator groups were examined as a measure of the gap between costs and reimbursements.^[16]^

Mean (standard deviation, SD), and median (interquartile range, 25%–75% [IQR]) were calculated for continuous variables, and counts and proportions for categorical variables. One-way ANOVA, Student *t* test or the Wilcoxon rank-sum test were used to examine the differences in continuous variables as appropriate, while the chi-square test was used for categorical variables comparisons.

To adjust for confounding, we developed generalized linear models (GLM) to compare the differences in the continuous outcomes of interest (LOS and costs) between groups. Since the continuous outcomes were positive and non-normal (skewed), we ran the GLMs with a logarithmic link and used a Gamma family distribution. Robust standard errors were derived based on the hospital of the patient encounter.^[17]^ For binary outcome models (mortality, 30-day readmission), a multilevel mixed-effects logistic regression model structure was used with hospitals of the patient encounters treated as random effects.

All inferences were two-tailed. Statistical significance was defined to be present at *α* = 0.05. Unless otherwise stated, the *P*-values are reported for comparisons between all 3 groups. Statistical analyses were performed using Stata/MP 15.1 for Windows for Windows (StataCorp, College Station, TX).

### 2.7 Ethics statement

Because this study used already existing publicly available fully de-identified retrospective data, it was exempt from ethics review.

## 3 Results

**Figure 1:**
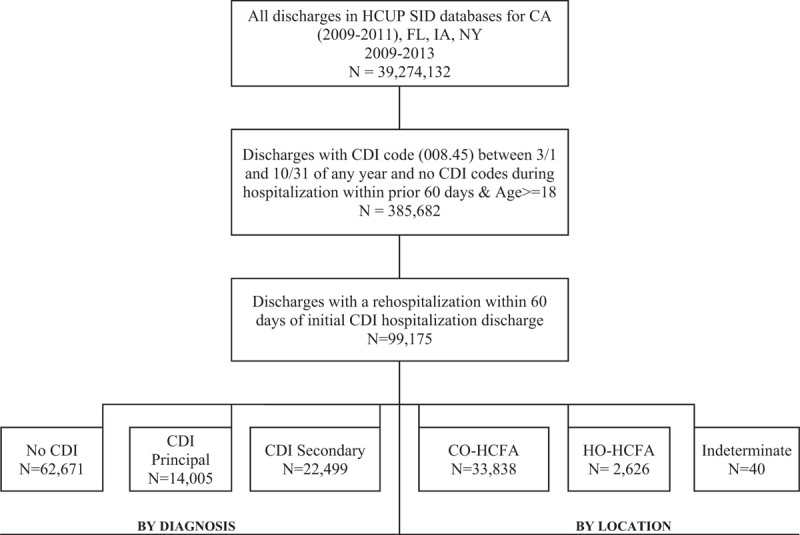
Enrollment chart. CDI = *C difficile* infection, CO = community-onset, HCFA = healthcare facility associated, HCUP = Health Care Utilization Project, HO = hospital-onset, SID = State Inpatient Database.

Among 385,682 initial CDI hospitalizations identified between years 2009 and 2013 in the 4 states examined, 99,175 (25.7%) required a rehospitalization (Fig. 1). Of them, 36,504 (36.8%) had a rCDI (14,005 [14.1%] as PrCDI), while the majority, 62,671 (63.2%), had no code for CDI. In each group, Florida contributed the plurality of admissions, followed by New York, California, and Iowa (Table 1 ). Nearly all rCDI were present on admission (POA) (99.7% PrCDI, 88.4% SrCDI) (Table 1 ), and nearly all were CO-HCFA (33,838, 92.7%) (Fig. 1).

**Table 1:**
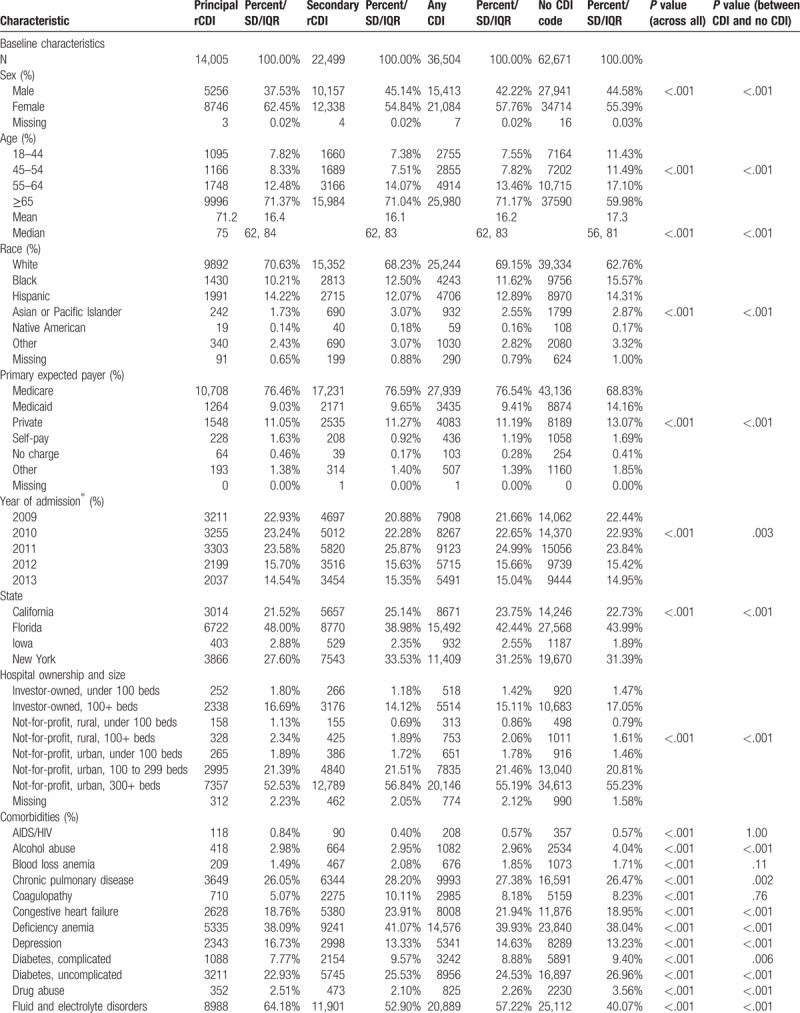
Baseline characteristics, admission, sources, and hospital events.

**Table 1 (Continued):**
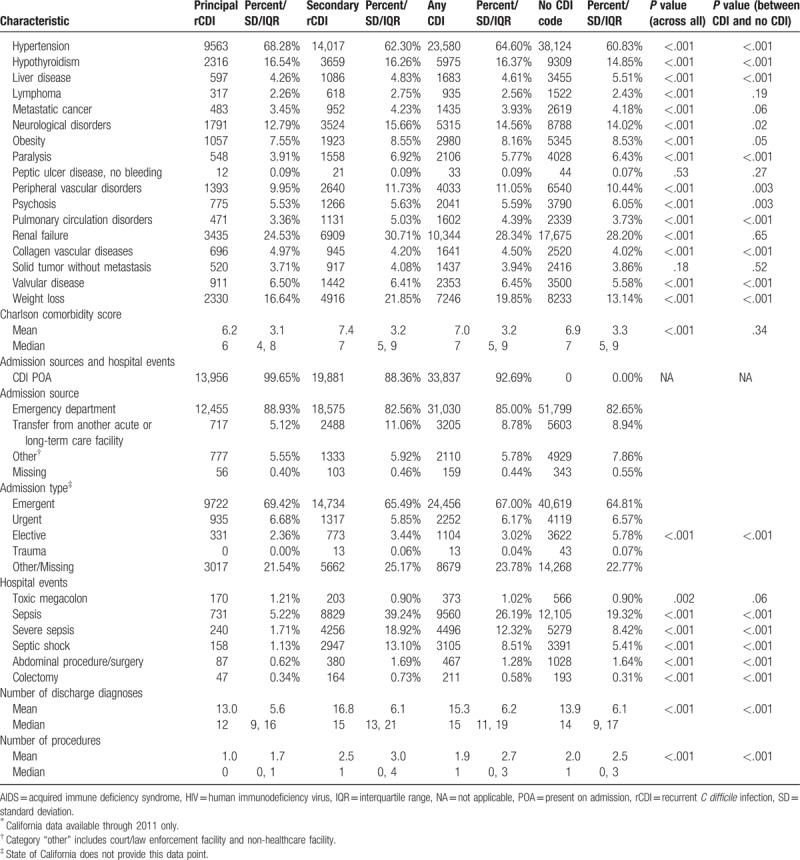
Baseline characteristics, admission, sources, and hospital events.

While patients with PrCDI and SrCDI were older than the non-CDI subjects, SrCDI and non-CDI groups had fewer females than PrCDI group (Table 1 ). PrCDI group had the lowest and SrCDI's the highest comorbidity burden, with non-CDI falling in-between (Table 1 ). Sepsis prevalence was nearly 8 times higher in SrCDI (39.2%) than in PrCDI (5.2%), and twice that in non-CDI (19.3%, *P* < .001). The prevalence of severe sepsis and septic shock followed a similar pattern (Table 1 ).

Both unadjusted hospital mortality and costs were highest in the SrCDI group and lowest in PrCDI (Supplement Table 1). LOS, on the other hand, was similar in PrCDI and non-CDI groups and both were lower than in SrCDI (Supplement Table 1). Interestingly, 30-day readmissions were highest among non-CDI and lowest in PrCDI, though in all groups the rate was over 30% (Supplement Table 1).

In the adjusted analyses, comparing PrCDI and SrCDI outcomes to the reference group of non-CDI, PrCDI (odds ratio [OR] 0.52; 95% confidence interval [CI] 0.46, 0.58) and SrCDI (0.80; 95%CI 0.75, 0.85) were associated with a lower risk of death than non-CDI (Table 2). However, among both groups of rCDI patients we noted excess LOS (PrCDI 1.8 days; 95% CI 1.7, 2.0 and SrCDI 1.4 days; 95% CI 1.3, 1.5) and costs (PrCDI $1399; 95% CI $858, $1939 and SrCDI $2809; 95% CI $2307, $3311) relative to the non-CDI group (Table 2). Similar to mortality, however, adjusted 30-day readmission risk was lower in PrCDI (OR = 0.84; 95% CI 0.80, 0.88) and SrCDI (OR = 0.97; 95% CI 0.94, 1.01) than non-CDI (Table 2).

**Table 2:**
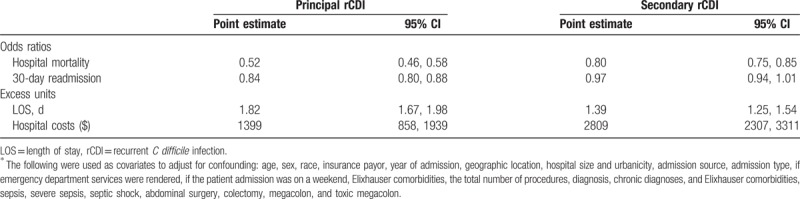
Adjusted outcomes for principal and secondary rCDI relative to no rCDI^∗^.

Among the top 5 DRGs, gastrointestinal disorders predominated in PrCDI, and sepsis did in SrCDI and non-CDI (Table 3). The mean gap between hospital costs and DRG-reimbursements was highest in SrCDI ($13,803) and lowest in PrCDI ($4881) (Table 3).

**Table 3:**
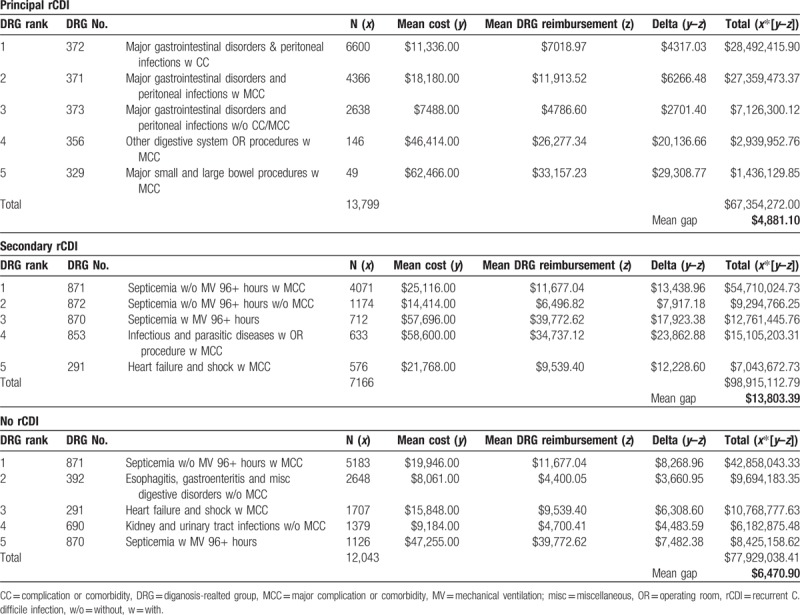
Reimbursement gap.

## 4 Discussion

We have demonstrated that in a cohort of patients who have survived an initial hospitalization with a CDI, a full one-quarter gets readmitted within 60 days, of whom over 1/3 has a diagnosis of rCDI during that readmission. While only approximately one-third of all rCDI is PrCDI, nearly all are considered to be POA. We further found that those patients who are readmitted with PrCDI are different demographically and clinically from those with SrCDI and those without CDI. Indeed, among those 3 groups, patients who are rehospitalized with rCDI as a secondary diagnosis tend to exhibit the highest severity of illness. Nevertheless, both SrCDI and PrCDI patients have a lower adjusted odds of hospital death and 30-day readmission than non-rCDI patients. However, even after adjustment, both PrCDI and SrCDI are associated with increased hospital LOS and costs relative to non-rCDI hospitalizations. Notably, SrCDI carries the highest mean reimbursement gap among the 3 groups examined; nearly $14,000 per hospitalization.

The difference in hospital mortality between those with SrCDI (11.5%) and PrCDI (2.9%) implies that, even in the setting of rCDI, some other factors are likely driving outcomes. For example, sepsis, present in 18.9% of SrCDI as compared with 1.7% of PrCDI, could explain much of the difference. Alternatively, it is possible that this reflects the limited space for billing codes in the HCUP databases. Hence there may be a coding bias wherein those patients with greater disease burden may be less likely to have a CDI code noted if the coder deems it to be less severe and hence less important than other conditions. This observation may further explain why our findings differ from those of others.^[8]^

At the same time, we observed a similar relationship between rCDI and death in a cohort of Medicare recipients whose initial CDI hospitalization required discharge to a chronic care facility.^[4]^ In that study, approximately 25% of patients with and without rCDI died within 60 days of rCDI onset. The lower overall rates of death in the current analysis likely reflect a younger population than in other studies, coupled with a shorter observation period limited to the acute hospitalization. In contrast, Olsen et al^[8]^ reported a 33% rise in the adjusted relative risk of death within 180 days in association with rCDI.

One novel aspect of our study is its exploration into the potential differences in outcomes between patients admitted to the hospital specifically to treat the recurrence itself (PrCDI), as opposed to where rCDI is a secondary reason for admission (SrCDI). This distinction appears important, as the outcomes diverge substantially. Therefore, different therapeutic strategies may be necessary in the 2 groups to optimize those outcomes. It is not surprising, for example, that mortality among PrCDI patients is substantially lower than SrCDI, since SrCDI patients were far more likely to be admitted with such high-risk conditions as sepsis.

Ours is not the first study to demonstrate the excess costs and LOS in association with rCDI. Over a 180-day period following index CDI, Olsen et al^[9]^ found an excess of 11 hospital days among those with rCDI over those without, and the number of readmissions per patient was 1.72 versus 0.81 for the groups, respectively. In a Medicare cohort of older subjects, rCDI resulted in an excess of 20 days of acute hospitalization.^[4]^ Although the number of excess days in the hospital is lower in the current study than in each of the prior 2, this again is likely due to the shorter observation period and a younger population we studied. Nevertheless, despite having no impact on in-hospital mortality, both PrCDI and SrCDI contributed substantially to an increase in the LOS. More importantly, this excess LOS translated into significant excess costs, consistent with prior reports.^[4,11]^

To the best of our knowledge, ours is the first study to examine 2 additional outcomes in the setting of rCDI hospitalization, namely, 30-day readmission rates and the reimbursement gap. These outcomes carry a particular importance to hospitals whose finances are already strained. It should be reassuring to both patients and institutions that the 30-day readmission rates do not appear to be higher with than without rCDI. However, rCDI admissions pose a major financial challenge since in both cases, patients hospitalized either with or for rCDI consume resources well beyond amounts covered by third party payors. This is particularly true in the instance of SrCDI, where we estimate each hospitalization nets the institution a $14,000 loss per admission. While the SrCDI-associated reimbursement gap is the largest, the other 2 groups’ reimbursement shortfalls are also substantial. Even when comparing similar DRG categories, such as 870 and 871 (sepsis) between SrCDI and no rCDI, there is almost twice the magnitude of this gap, defining these values may help hospitals shed a quantitative light on discrepancies they may encounter.

Our study has a number of limitations. As a retrospective study it is subject to a number of biases, most notably selection bias. To mitigate this we developed a priori inclusion criteria. Because we used administrative coding to identify rCDI, there is a possibility for misclassification. However, using ICD-9-CM codes to identify incident CDI is well validated in the hospitalized population.^[18]^ Still, while this method of defining rCDI in the hospital has been used previously, it has not been widely validated.^[4]^ In fact, the presence of this code may or may not signify the actual presence of active CDI. Wen et al^[19]^ found a relatively low specificity of a combination of ICD-9 codes, stool testing procedure codes, and CDI treatment codes for recurrent CDI. This raises the possibility that at least in a proportion of those identified, rCDI reflects the patient having a documented history of CDI, rather than an active episode. However, this type of misclassification is likely to drive our effect estimates closer to the null and thus mute any differences that exist between comparator groups. This may in part account for our inability to find an association between rCDI and either mortality or readmission rates.

Confounding is an issue in observational studies. Although we dealt with the possibility of confounding by deriving regression models to estimate the effect of rCDI on the outcomes of interest using a large number of additional covariates, the possibility of residual confounding remains. At the same time, our analysis may also suffer from overadjustment.^[20]^ In particular, it is possible that sepsis, for example, at least in some cases, was an intermediary between rCDI and hospital outcomes. In such a case examining it as a confounder may have shifted our effect estimates toward the null, implying that the actual impact of rCDI on the outcomes is likely to be greater than what we found. The risk of this is highest in the SrCDI group, where the prevalence of sepsis was far greater than in PrCDI.

These limitations notwithstanding, our study has a number of strengths that lend credibility to our results. Since we relied on a large geographically representative sample of rCDI discharges, our results are broadly generalizable to US hospitals. We applied rigorous statistical methodologies to derive attributable outcomes. The novel aspects of our study—examining PrCDI and SrCDI separately, computing rCDI's impact on 30-day readmission rates, and the calculation of the reimbursement gap—augment the usefulness and relevance of our data in underscoring the financial pressures that should drive institutions to target intensive preventive efforts.

In summary, a rehospitalization following an index CDI admission is a common occurrence, and a substantial proportion of those readmissions involve rCDI. PrCDI patients differ from those with SrCDI, the latter more similar to those rehospitalized without rCDI, but more severely ill and suffering worse economic outcomes. Although we found no evidence of an increase in mortality or 30-day readmission due to PrCDI or SrCDI relative to no CDI, both increase the LOS and hospital costs. Importantly, all groups, and SrCDI in particular, incurred a substantial deficit in reimbursements when compared with the expenditures, thus suggesting that more efficient models of care may be needed for these patients.

## Acknowledgments

No persons other than the authors participated in data retrieval, analysis, interpretation, or manuscript preparation.

## Author contributions

MDZ has participated in study design, results interpretation, manuscript drafting, and revision.

BHN has participated in analysis, and manuscript review and revision. Dr Nathanson had full access to all of the data in the study and takes responsibility for the integrity of the data and the accuracy of the data analysis.

SWM has participated in study design, analysis, and manuscript review and revision.

JJH has participated in study design, analysis, results interpretation, manuscript drafting and revision.

AFS has participated in study design, results interpretation, manuscript drafting and revision.

**Conceptualization:** Marya D. Zilberberg, Brian H. Nathanson, Stephen W. Marcella, John Jay Hawkshead, Andrew F. Shorr.

**Data curation:** Brian H. Nathanson.

**Formal analysis:** Brian H. Nathanson.

**Funding acquisition:** Marya D. Zilberberg.

**Investigation:** Marya D. Zilberberg, Andrew F. Shorr.

**Methodology:** Marya D. Zilberberg, Brian H. Nathanson, Stephen W. Marcella, Andrew F. Shorr.

**Project administration:** Marya D. Zilberberg.

**Resources:** Marya D. Zilberberg, Stephen W. Marcella, John Jay Hawkshead.

**Supervision:** Marya D. Zilberberg.

**Validation:** Marya D. Zilberberg.

**Writing – original draft:** Marya D. Zilberberg.

**Writing – review & editing:** Marya D. Zilberberg, Brian H. Nathanson, Stephen W. Marcella, John Jay Hawkshead, Andrew F. Shorr.

## Supplementary Material

Supplemental Digital Content
