# Supplementary material for: Hospital readmission with Clostridium difficile infection as a secondary diagnosis is associated with worsened outcomes and greater revenue loss relative to principal diagnosis: A retrospective cohort study
Source: Medicine (Baltimore). 2018 Sep 7;97(36):e12212. doi: 10.1097/MD.0000000000012212 (PMC6133633; doi:10.1097/MD.0000000000012212)
Supplement: Supplemental Digital Content [file medi-97-e12212-s001.docx]

**Supplemental material**

**Data source description**

State Inpatient Databases (SID) is a part of the Health Care Utilization Project (HCUP) administered by the Agency for Healthcare Research and Quality (AHRQ). Our data derived from four geographically diverse US states (California [only available for years 2009-2011], Florida, Iowa and New York) for years 2009-2013 (1). The 48 states that participate in SID capture data on approximately 97% of all discharges in the US. While it is not possible to identify individual readmissions in the National Inpatient Sample (NIS) database, this is feasible in some of the SIDs due to the way individual patients are tracked within selected states, provided those readmissions occurred within the same state and year as the index hospitalization.

The SID databases include data on patient demographics, diagnoses and procedures, admission and discharge sources, in-hospital mortality, as well as hospital charges and length of stay for each discharge. Additional data files, linkable to discharges in the SID database, provide data on hospital characteristics, illness severity measures, and cost-to-charge conversion coefficients for each individual institution in the database.

The four states representing each of the four Census Bureau regions of the US were chosen because their geographic diversity increases the generalizability of our findings (2).

**rCDI categories definitions**

Community-onset healthcare facility associated (CO-HCFA) rCDI was defined by a CDI code in the “present on admission” (POA) field when available, or a CDI code as the principal diagnosis if POA was not available, and the patient was transferred from another healthcare facility or had evidence of admission to another hospital, skilled nursing facility (SNF) or a nursing home (NH) within 4 weeks of the index admission date (3). Hospital-onset healthcare facility associated (HO-HCFA) rCDI was defined as no CDI code POA or, if POA was unavailable, CDI diagnosis was secondary. An episode of rCDI was considered indeterminate when a CDI was POA or, if POA was unavailable, there was principal CDI diagnosis along with evidence of admission to another hospital, SNF or a NH within 4-12 weeks of the index admission date (4). Because rCDI was defined by a recurrence following a prior hospitalization within 60 days, community-acquired (CA) infection was excluded from the study.

**Supplement Table 1. Unadjusted hospital outcomes**

| **Characteristic** | **Principal rCDI** | **Percent** | **Secondary rCDI** | **Percent** | **Any rCDI** | **Percent** | **No CDI code** | **Percent** | ***p* value (across all)** | ***p* value (between CDI and no CDI)** |
| --- | --- | --- | --- | --- | --- | --- | --- | --- | --- | --- |
| N | 14005 | 100.00% | 22499 | 100.00% | 36504 | 100.00% | 62671 | 100.00% |  |  |
|  |  |  |  |  |  |  |  |  |  |  |
| Hospital mortality | 402 | 2.87% | 2584 | 11.48% | 2986 | 8.18% | 4865 | 7.76% |  |  |
|  |  |  |  |  |  |  |  |  |  |  |
| Length of stay (days) |  |  |  |  |  |  |  |  |  |  |
| Mean | 7.3 |  | 10.6 |  | 9.4 |  | 7.3 |  |  |  |
| ± SD | 7.0 |  | 11.6 |  | 10.2 |  | 8.2 |  | <0.001 | <0.001 |
| Median | 6 |  | 7 |  | 6 |  | 5 |  |  |  |
| IQR | 4,9 |  | 4, 13 |  | 4, 11 |  | 3,9 |  |  |  |
|  |  |  |  |  |  |  |  |  |  |  |
| Total charges ($) |  |  |  |  |  |  |  |  |  |  |
| Mean | 47345 |  | 90486 |  | 73793 |  | 34218 |  |  |  |
| ± SD | 60090 |  | 129073 |  | 109781 |  | 43507 |  | <0.001 | <0.001 |
| Median | 31910 |  | 51592 |  | 41839 |  | 22820 |  |  |  |
| IQR | 18823, 54323 |  | 26672, 104138 |  | 22523, 81298 |  | 13252, 39497 |  |  |  |
|  |  |  |  |  |  |  |  |  |  |  |
| Total costs ($) |  |  |  |  |  |  |  |  |  |  |
| Mean | 13748 |  | 26929 |  | 21838 |  | 17825 |  |  |  |
| ± SD | 18317 |  | 39569 |  | 33641 |  | 26545 |  | <0.001 | <0.001 |
| Median | 8962 |  | 15246 |  | 12130 |  | 10384 |  |  |  |
| IQR | 5420, 15585 |  | 7969, 30317 |  | 6627, 23867 |  | 5683, 19849 |  |  |  |
|  |  |  |  |  |  |  |  |  |  |  |
| 30-day readmission among survivors | 4214 | 30.98% | 6960 | 34.95% | 11174 | 33.34% | 21642 | 37.44% | <0.001 | <0.001 |

rCDI = recurrent *C. difficile* infection; SD = standard deviation; IQR = interquartile range

**References**

1. HCUP: Healthcare Cost and Utilization Project. Overview of State Inpatient Database (SID). Available at <https://www.hcup-us.ahrq.gov/sidoverview.jsp>; accessed January 26, 2018
2. Goodwin AJ, Rice DA, Simpson KN, Ford DW. Frequency, cost, and risk factors of readmissions among severe sepsis survivors. Crit Care Med 2015;43:738-46
3. Cohen SH, Gerding DN, Johnson S, Kelly CP, Loo VG, McDonald LC et al. Clinical practice guidelines for *Clostridium difficile* infection in adults: 2010 update by the society for healthcare epidemiology of America (SHEA) and the infectious diseases society of America (IDSA). Infect Control Hosp Epidemiol 2010;31:431-55
4. Pakyz AL, Patterson JA, Motzkus-Feagans C, Hohmann SF, Edmond MB, Lapane KL. Preformance of the present-on-admission indicator for *Clostridium difficile* infection. Infect Control Hosp Epodemiol 2015;36:838-40
